# Zinc deficiency impairs ischemia-induced angiogenesis

**DOI:** 10.1016/j.jvssci.2021.09.023

**Published:** 2021-12-08

**Authors:** Takuya Tsuruoka, Akio Kodama, Shukuro Yamaguchi, Tomohiro Masutomi, Akio Koyama, Toyoaki Murohara, Kimihiro Komori, Rei Shibata

**Affiliations:** a Division of Vascular and Endovascular Surgery, Department of Surgery, Nagoya University Graduate School of Medicine, Nagoya, Japan; b Department of Cardiology, Nagoya University Graduate School of Medicine, Nagoya, Japan; c Department of Advanced Cardiovascular Therapeutics, Nagoya University Graduate School of Medicine, Nagoya, Japan

**Keywords:** Angiogenesis, Ischemia, Zinc

## Abstract

**Objective:**

Zinc is an important essential trace metal involved in many physiologic functions, and its deficiency can affect the development of multiple organs, including the vasculature. However, clarity is lacking regarding the effects of zinc deficiency in the regulation of angiogenesis. We investigated the effects of zinc deficiency on the revascularization process through animal experiments and examined the relationship between the circulating zinc levels and tissue blood perfusion in patients with chronic limb-threatening ischemia (CLTI).

**Methods:**

Zinc-deficient mice and control wild-type mice had undergone surgery to create unilateral hindlimb ischemia. Next, we examined the relationship between the serum zinc levels and skin perfusion pressure (SPP) as an index of tissue blood perfusion in patients with CLTI. A total of 51 patients with CLTI who had been referred for de novo revascularization for CLTI due to arteriosclerosis obliterans at our hospital from May 2012 to March 2016 were enrolled.

**Results:**

The zinc-deficient mice showed a significant reduction in blood flow recovery rates in the ischemic limb and capillary density in the ischemic adductor muscle fibers compared with the control wild-type mice. The zinc-deficient mice also showed increased reactive oxygen species production after hindlimb ischemia. Nicotinamide adenine dinucleotide phosphate oxidase inhibitors ameliorated the zinc deficient-induced impairment of revascularization. The serum zinc levels were positively associated with the SPP in the CLTI patients. Multivariate regression analysis also revealed that the serum zinc levels were significantly correlated with the SPP in patients with CLTI.

**Conclusions:**

Zinc deficiency impaired the rate of ischemia-induced revascularization through enhanced oxidative stress rates, suggesting that nutritional management for zinc sufficiency could be useful in CLTI prevention and treatment.

## Clinical Relevance

In the present study, we investigated the effects of zinc deficiency on angiogenesis. We found that zinc deficiency impaired the rate of ischemia-induced revascularization through enhanced oxidative stress rates in animal model. In addition, the skin perfusion pressures were positively associated with the serum zinc levels in patients with chronic limb-threatening ischemia. Thus, the intake of zinc could be useful for the prevention and/or treatment of ischemic limb disease. Circulating zinc levels could be a useful marker for the assessment of atherosclerosis-based vascular disease such as limb ischemia. Possibly, nutritional improvement by zinc intake could lead to the prevention and treatment of ischemic vascular disease.

**Article Highlights**

• **Type of Research:** A single-center, retrospective, cohort study and animal experiments
• **Key Findings:** Zinc deficiency impaired the rate of ischemia-induced revascularization through enhanced oxidative stress rates in mice experiments. The serum zinc levels were also positively associated with the skin perfusion pressure in 51 patients with chronic limb-threatening ischemia who had been referred for de novo revascularization for chronic limb-threatening ischemia due to arteriosclerosis obliterans (*r* = 0.538; *P* < .001).
• **Take Home Message:** The intake of zinc could be useful for the prevention and/or treatment of ischemic limb disease. In addition, circulating zinc level could be a useful marker for the assessment of atherosclerosis-based vascular disease such as limb ischemia.

The incidence of peripheral arterial disease (PAD) has been increasing globally. In no less than one half of patients with PAD who have chronic limb-threatening ischemia (CLTI), the affected limbs had been amputated, which impairs patients' quality of life and lifespan.^1^ PAD develops as a result of lifestyle changes, such as the lack of physical activity, overnutrition, and the dyshomeostasis of trace elements.^2^ Although the influence of selenium and copper on the development of atherosclerosis and heart disease has attracted a high degree of attention,^3^^,^^4^ the effects of trace metal deficiencies on this process is less well-defined. Information is lacking on the effects of trace elements, including zinc, on angiogenesis. Moreover, no specific signaling pathways associated with angiogenesis have been identified.

In recent years, the importance of zinc nutrition has gained global recognition.^5^ Zinc is an important essential trace metal required for many physiologic functions, including growth and reproduction. Zinc deficiency can affect the development of multiple organs, including the brain, lungs, kidneys, heart, and vasculature.^6^ Some of the major health effects of zinc deficiency include reduced growth rates and immune function suppression.^6^^,^^7^ Thus, zinc deficiency has been widely recognized as a leading risk factor for morbidity and mortality.

Regarding the development of cardiovascular disease, arterial pressure has been inversely correlated with serum zinc concentrations.^8^ Zinc deficiency that has been induced by zinc metabolism disturbances has been correlated with cardiovascular events.^6^^,^^7^ A high zinc intake provides protection against cardiovascular disease.^9^ Recently, we investigated the relationship between zinc deficiency and the clinical outcomes after bypass surgery for CLTI and found that zinc deficiency is associated with lower rates of limb salvage.^10^ The development of cardiovascular disease, including CLTI, has been associated with blood zinc levels, and the reduced angiogenic capacity is extremely involved in the pathology of CLTI.^11^ Therefore, we hypothesized that zinc might regulate angiogenesis in CLTI. Accordingly, we investigated the effects of zinc deficiency on ischemia-induced revascularization processes using a murine hindlimb ischemia model and also examined the relationship between the circulating zinc levels and skin perfusion pressure (SPP) as an index of tissue blood perfusion in patients with CLTI.

## Methods

### Materials

CD31 antibody was purchased from BD (Franklin Lakes, NJ). Dihydroethidium (DHE) and apocynin were purchased from Wako Pure Chemical (Osaka, Japan).

### Zinc-deficient mouse model and mouse model of limb blood perfusion

Wild-type (WT) and diet-induced, zinc-deficient mice on a C57/BL6 background were used in the present study. The institutional animal care and use committee at Nagoya University approved all the study protocols. Zinc deficiency was induced by the provision of ad libitum access to a zinc-deficient diet (0.01% zinc content; Oriental BioService, Inc, Kyoto, Japan) to 3-week-old mice for 7 weeks.^12^ Until the time of the experiment, the lean controls were fed a standard chow diet. The number of the zinc-deficient mice and control mice was 8 in each group. At the age of 10 weeks, the serum zinc levels of the mice were measured, and the mice had undergone unilateral hind limb surgery under anesthesia with sodium pentobarbital (administered intraperitoneally at 50 mg/kg). As reported previously, the entire left femoral artery and vein were surgically removed in this model.^13^^,^^14^ Every mouse was weighed weekly, and a tail-cuff pressure analysis system was used to determine the heart rate and systolic blood pressure with the mouse in the conscious state. In some experiments, the mice were treated with apocynin or a vehicle from the age of 3 weeks until sacrifice. Apocynin has the effect of inhibiting nicotinamide adenine dinucleotide phosphate (NADPH) oxidase activity and suppressing the production of reactive oxygen species (ROS). Apocynin powder was added to hot (∼60°C) sterile water. The water was allowed to cool to room temperature before being provided to the mice. The concentration of apocynin was 2 mg/mL in water. The conversion to mg/kg was estimated by the average daily water intake of ∼4 mL per mouse, resulting in a dose of ∼300 mg/kg/d.^15^

### Laser Doppler blood flow analysis

The hindlimb blood flow was measured using a laser Doppler blood flow (LDBF) analyzer (Moor LDI; Moor Instruments Inc, Wilmington, Del) immediately before surgery and on postoperative days 3, 7, 14, and 28. LDBF analysis was performed on the legs and feet. Changes in the laser frequency were represented in the blood flow using different color pixels. After scanning, the stored images were analyzed for blood flow quantification. To avoid the data from varying by the ambient light and temperature, the ratio of the left (ischemic) to right (nonischemic) LDBF was used to express the hindlimb blood flow.^13^^,^^14^

### Capillary density analysis

The capillary density in the adductor muscle was analyzed to obtain specific evidence on the vascularity at the microcirculation level. On postoperative day 28, we acquired tissue samples from the ischemic thigh adductor skeletal muscles. Frozen tissue slices (7 μm in thickness) were prepared and stained with CD31 and then treated with fluorescein isothiocyanate-conjugated secondary antibody for the detection of CD31. The signals were detected and analyzed using fluorescence microscopy. To determine the presence of capillary endothelial cells, in each tissue block, we examined 15 random microscopic fields from 3 different sections. The number of capillary endothelial cells per field was determined as the capillary density.^13^

### In situ DHE staining

The superoxide production rate was evaluated by in situ DHE staining. We placed the samples in optimal cutting temperature compound, which were then snap-frozen in liquid nitrogen. Tissue slices (7 μm in thickness) were arranged and incubated with DHE in phosphate-buffered saline (10 mmol/L) for 30 minutes at room temperature in a dark and humidified container. The reaction of superoxide with ethidium bromide oxidizes DHE, which binds to the DNA in the nuclei and emits red fluorescence. Fluorescence microscopy was used to detect and analyze the signals. The excitation wavelength was 488 nm, and a 568-nm, long-pass filter was used to detect emission fluorescence.^16^

### Biomarker analysis

The serum zinc levels in the mouse blood samples were measured using enzymatic kits (Metallogenics, Co, Ltd, Chiba, Japan). This assay system measures both free and protein-bound zinc.^17^ Human blood samples were obtained from the patients before surgery. The complete blood counts were measured using a Sysmex XE-5000 hematology analyzer (Sysmex, Kobe, Japan). Biochemical data, including those pertaining to zinc, were measured using a LABOSPECT 008 automatic analyzer (Hitachi Co, Tokyo, Japan).

A free radical analytic system was used to measure the derivatives of reactive oxidative metabolites (d-ROMs), according to the manufacturer's instructions. The d-ROMs test is grounded on the idea that the amount of organic hydroperoxides in the blood and that of free radicals from which they are formed, are related. Thus, the hydroperoxides react with the transition metal when the sample is dissolved in an acidic buffer. Using a spectrophotometer, the concentrations of these persistent species can be determined at 505 nm. The d-ROMs are expressed in Carratelli units, where 1 Carratelli unit is equivalent to 0.8 mg/L of hydrogen peroxide.^16^ The serum nitrotyrosine levels were measured using a nitrotyrosine enzyme-linked immunosorbent assay kit (StressMarq Biosciences Inc, Victoria, British Columbia, Canada) according to the manufacturer's instruction.

### Measurement of messenger RNA

Total RNA was extracted from the muscle tissues using an RNeasy lipid tissue mini kit (Qiagen, Inc, Hilden, Germany) according to the manufacturer's protocols. Complementary DNA from 500 ng of total RNA was synthesized by reverse transcription using ReverTra Ace reverse transcription (RT)-quantitative polymerase chain reaction (qPCR) master mix (Toyobo Life Science, Osaka, Japan) according to the manufacturer's instructions. qRT-PCR analysis was performed with a CFX-96 system using THUNDERBIRD qPCR mix (Toyobo Life Science) as a double-stranded DNA-specific dye, according to the manufacturer's instructions (Bio-Rad, Hercules, Calif).^18^ The primers were designed as follows: 5′-CAGGCTGCTGTAACGATGAA-3′ and 5′-AATGCTTTCTCCGCTCTGAA-3′ for mouse vascular endothelial growth factor (VEGF); 5′-TTGGGTCAGCACTGGCTCTG-3′ and 5′-TGGCGGTGTGCAGTGCTATC-3′ for mouse gp91^phox^; 5′-GGCCATTGCCAGTGTGATCTA-3′ and 5′-TGCTTGATGGTGCCTCCAA-3′ for mouse p22^phox^; 5′-GATGTTCCCCATTGAGGCCG-3′ and 5′-GTTTCAGGTCATCAGGCCGC-3′ for mouse p47^phox^; 5′-CTGGCTGAGGCCATCAGACT-3′ and 5′-AGGCCACTGCAGAGTGCTTG-3′ for mouse p67^phox^; 5′-ATGGTGAAGGTCGGTGTG-3′, and 5′-ACCAGTGGATGCAGGGAT-3′ for glyceraldehyde 3-phosphate dehydrogenase. The expression levels of the examined transcripts were compared with those of glyceraldehyde 3-phosphate dehydrogenase and normalized to the mean value of the controls.

### Clinical study

The data from 51 consecutive patients with CLTI who had been referred for de novo revascularization for CLTI due to arteriosclerosis obliterans at our hospital from May 2012 to March 2016 were retrospectively analyzed. Blood samples obtained from the patients before surgery were used to measure the biochemical data, including those pertaining to zinc. The present study was conducted in compliance with the principles of the Declaration of Helsinki. The Nagoya University School of Medicine institutional review board approved the present study (approval no. 2019-0185), and all the patients had provided written informed consent for data collection.

CLTI was defined as resting pain that had lasted >2 weeks and/or tissue loss or gangrene in accordance with Inter-Society Consensus for the Management of Peripheral Arterial Disease guidelines.^19^ A medical history was obtained to document the medical history, medications, and comorbid disease. The body mass index was calculated as the ratio of the weight to the height squared. Diabetes mellitus was defined according to the World Health Organization criteria. Hypertension was defined as a systolic blood pressure ≥140 mm Hg or diastolic blood pressure ≥90 mm Hg on repeated measurements or the receipt of antihypertensive treatment. Dyslipidemia was defined as a total cholesterol level of ≥220 mg/dL or a triglyceride level ≥150 mg/dL or the receipt of lipid-lowering therapy. The definition of coronary artery disease (CAD) was a history of any revascularization of the coronary arteries. The definition of cardiovascular disease was a history of stroke, cerebral hemorrhage, and/or any revascularization of the carotid arteries.

In the present study, we measured the SPP as an index of tissue blood perfusion. The SPP was measured with the patient in the supine position on a bed at room temperature using a laser Doppler probe (SensiLase PAD3000 Doppler Waveform Analyzer; Kaneka Medix Corp, Osaka, Japan). A SPP <40 mm Hg with tissue loss or gangrene or SPP <30 mm Hg with resting pain was considered to indicate CLTI.^20^ The SPP values were obtained by trained medical technologists from the patients before surgery.

### Statistical analysis

All statistical analyses were performed using PASW Statistics, version 27, software (IBM Corp, Armonk, NY). The data are expressed as the mean ± standard deviation. Analysis of variance, followed by Tukey's honestly significant difference tests and unpaired Student's *t* tests, were used for statistical analysis. For the identification of the covariates associated with the SPP, Spearman's rank order correlation was conducted. Variables with *P* < .05 on univariate analysis were incorporated into the multivariable model. Multiple linear regression was calculated to predict the SPP from the variables. In all the analyses, *P* < .05 was considered statistically significant.

## Results

### Zinc-deficient mice showed a reduced rate of ischemia-induced revascularization

**Supplementary Fig 1:**
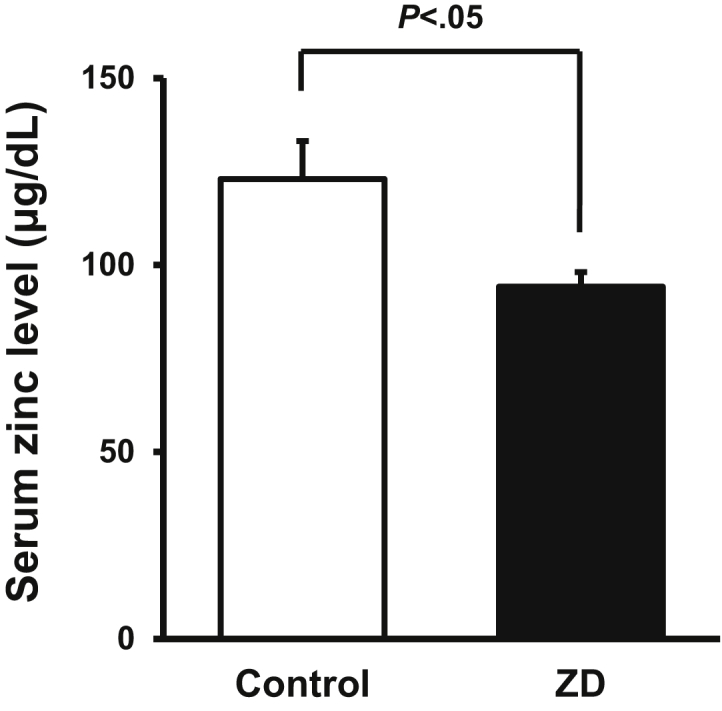
Bar graph showing serum zinc levels in control and zinc-deficient (ZD) mice.

To assess the effects of zinc deficiency on the revascularization process in response to ischemia, the WT mice treated with or without a zinc-deficient diet were subjected to unilateral hindlimb ischemia. All the mice survived after the surgical induction of unilateral hindlimb ischemia. All the mice also appeared healthy during the follow-up period. The body weight and blood pressure did not differ between the zinc-deficient mice and control mice. The skin conditions remained unchanged, and no behavioral problems were observed. The serum zinc concentration was 94.2 ± 3.8 μg/dL in the zinc-deficient mice and 123.0 ± 10.2 μg/dL in the control WT mice at 10 weeks of age (*P* < .05; Supplementary Fig 1, online only).

**Fig 1:**
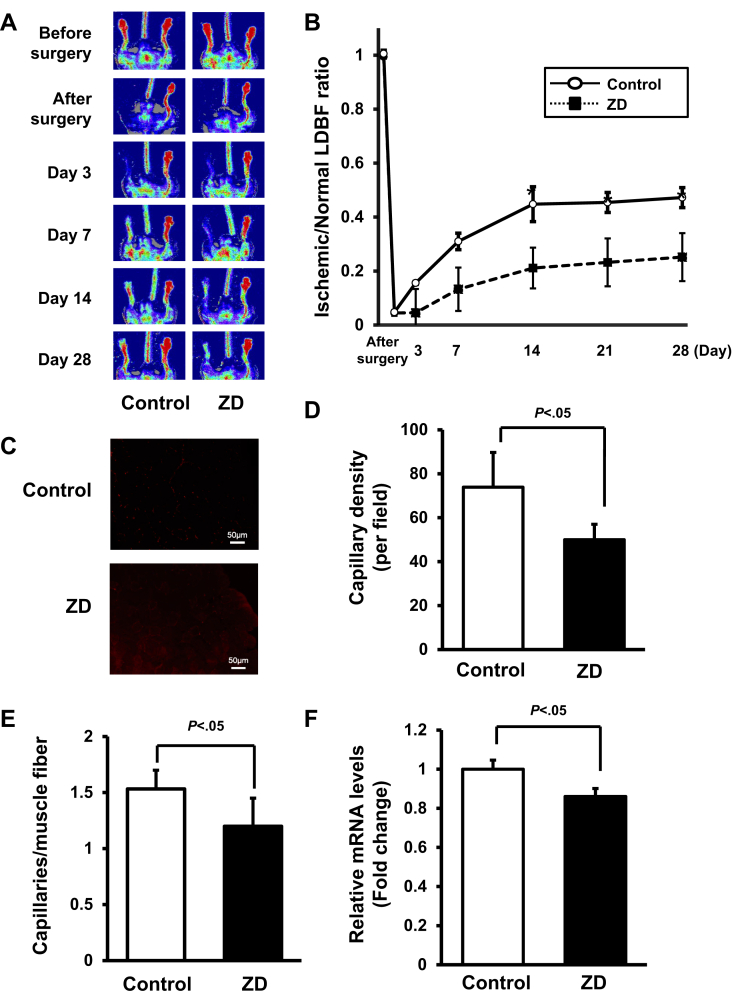
Zinc-deficient (*ZD*) mice showing reductions in the rates of perfusion recovery and capillary vessel formation in ischemic limbs. **A,** Representative laser Doppler blood flow (LDBF) images of the ischemic limb of ZD mice or control wild-type (WT) mice. A low perfusion signal (*dark blue*) was observed in the ischemic hindlimb of ZD mice. In contrast, a high perfusion signal (*red*) was detected in the control WT mice at postoperative days 14, 21, and 28. **B,** Quantitative analysis of the ischemic/nonischemic LDBF ratio in the ZD mice or control WT mice before surgery and at different points after surgery. Results are presented as the mean ± standard deviation (n = 8 in each group). ∗*P* < .05 vs ZD mice. **C,** Fluorescence staining of ischemic tissues with anti-CD31 monoclonal antibody (*red*) on postoperative day 28. **D** and **E,** Quantitative analysis of capillary density in ZD mice or control WT mice on postoperative day 28. Capillary density expressed as the number of capillaries per high power field (×400) (**Left**) and capillaries per muscle fiber (**Right**). **F,** Messenger RNA (mRNA) levels of vascular endothelial growth factor (VEGF) in the ischemic muscle in the ZD mice or control WT mice on postoperative day 28. mRNA levels were measured using the real-time polymerase chain reaction (PCR) method (n = 8 in each group). All results were normalized to glyceraldehyde 3-phosphate dehydrogenase. ∗*P* < .01 vs control. Results presented as mean ± standard deviation (n = 8 in each group).

We performed LDBF analysis before surgery and on postoperative days 3, 7, 14, 21, and 28 for the evaluation of the perfusion recovery rate of the control and zinc-deficient mice after ligation of the femoral artery. Representative images of the blood perfusion measured by LDBF are shown in Fig 1, *A*. In the control mice, the hindlimb blood flow perfusion rate showed a steep decrease after surgery and had remained impaired for 3 days. By day 7, it had increased to 25% to 35% of that in the nonischemic limb, and by day 28, it had ultimately returned to 45% to 55% (Fig 1, *B*). In contrast to the blood flow in the control mice, the blood flow in the ischemic hindlimb was remarkably reduced in the zinc-deficient mice compared with that in the nonischemic hindlimb on postoperative days 7, 14, 21, and 28 (*P* < .05).

**Supplementary Fig 2:**
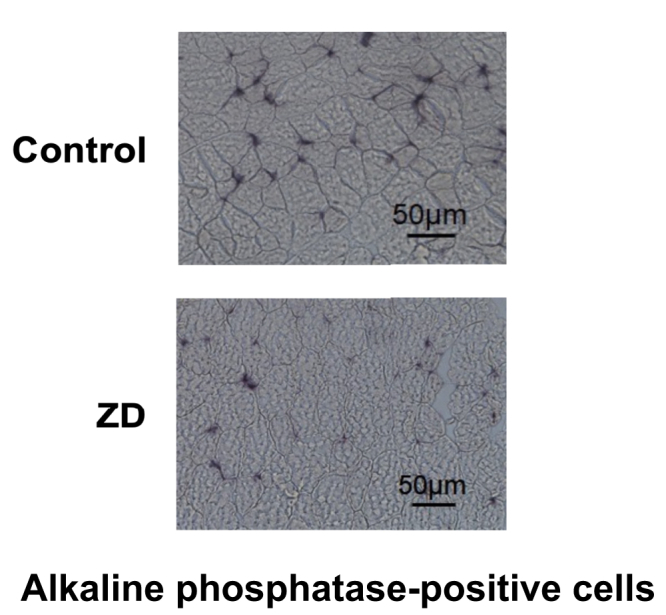
CD31 immunostaining results showing alkaline phosphatase-positive cells. ZD, Zinc deficiency.

The capillary density was measured in the ischemic tissues, and the degree of blood flow recovery at the microcirculatory level was evaluated by staining with anti-CD31 antibody. Representative photomicrographs of histologic sections stained with anti-CD31 antibody are shown in Fig 1, *C*. Quantitative analysis disclosed a significant reduction in the capillary density in the zinc-deficient mice compared with that in the control mice on postoperative day 28 (Fig 1, *D and E*). Furthermore, we assessed the capillary density using alkaline phosphatase staining. As shown by the CD31 immunostaining results, the counts of the alkaline phosphatase–positive cells were lower in the zinc-deficient mice than in the control mice (Supplementary Fig 2, online only).

In addition, we measured the expression of VEGF messenger RNA (mRNA) as one of the angiogenic factors in the ischemic muscle in the zinc-deficient and control mice on day 28 after hindlimb surgery using real-time PCR. The zinc-deficient mice showed lower VEGF mRNA levels compared with the levels in the control mice (Fig 1, *F*).

### Increased oxidative damage in zinc-deficient mice subjected to hindlimb ischemia

**Fig 2:**
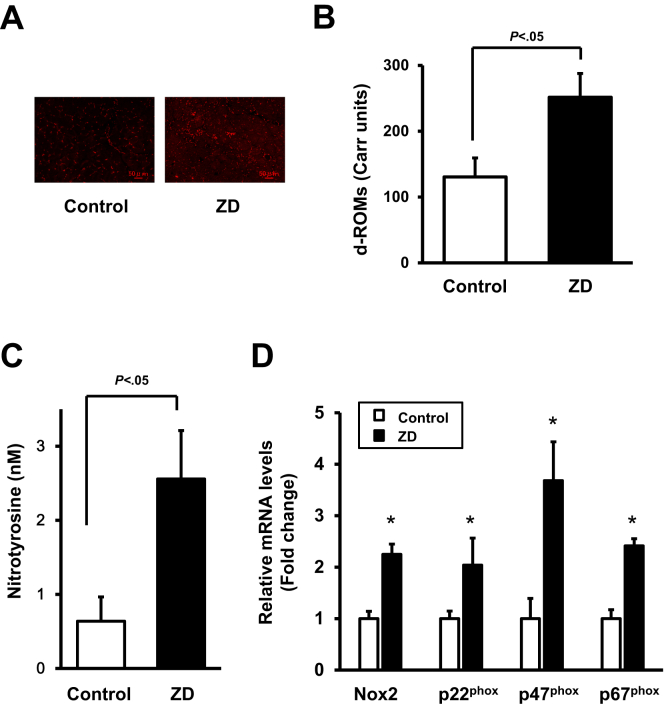
Zinc deficiency (*ZD*) increased the rate of oxidative damage in ischemic muscles. **A,** Production of reactive oxygen species (ROS) was evaluated by immunostaining with dihydroethidium (DHE) on postoperative day 28 (×400 for each field; *red*). Serum levels of the derivatives of reactive oxygen metabolites (d-ROMs; **B**) and nitrotyrosine **(C)** in the ZD mice or control wild-type (WT) mice after hindlimb surgery (n = 8 in each group). **D,** Messenger RNA (mRNA) levels of Nox2, p22^phox^, p47^phox^, and p67^phox^ in the ischemic muscle in the ZD mice or control WT mice on postoperative day 28. mRNA levels were measured using the real-time polymerase chain reaction (PCR) method (n = 8 in each group). All results were normalized to glyceraldehyde 3-phosphate dehydrogenase. ∗*P* < .01 vs control.

To examine the production of reactive oxygen species (ROS) in the ischemic muscle, DHE staining was performed and analyzed using fluorescence microscopy. Compared with the control mice, ROS production was significantly increased in the adductor muscle of the zinc-deficient mice (Fig 2, *A*).

Next, at 4 weeks after hindlimb ischemia surgery, we measured the serum levels of d-ROMs and nitrotyrosine, two indicators of oxidative stress. The serum d-ROMs and nitrotyrosine levels were significantly greater in the zinc-deficient mice than those in the control mice (Fig 2, *B and C*).

Furthermore, the mRNA levels of the NADPH oxidase ingredients in the ischemic muscle in the zinc-deficient and control mice on day 28 after hindlimb surgery were quantified using real-time PCRs. Higher expression of NADPH oxidase components, including Nox2, p22^phox^, p47^phox^, and p67^phox^, were confirmed in the zinc-deficient mice compared with the control mice (Fig 2, *D*). Thus, zinc deficiency impaired the rate of ischemia-induced angiogenesis, which is accompanied by excessive ROS production.

### NADPH oxidase inhibitors restore ischemia-induced angiogenesis in zinc-deficient mice

**Fig 3:**
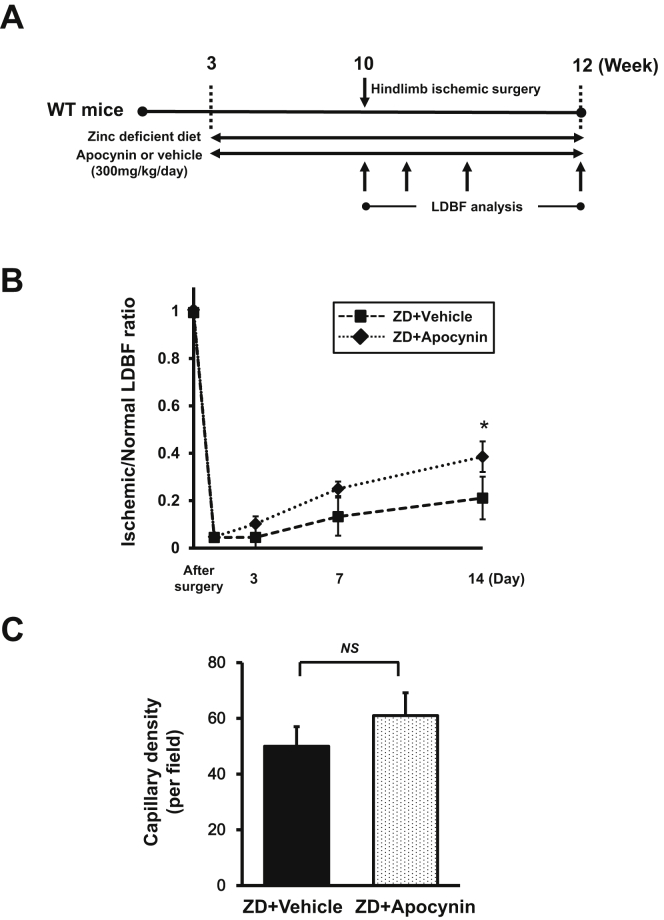
Nicotinamide adenine dinucleotide phosphate (NADPH) oxidase inhibitor, apocynin, restored ischemia-induced angiogenesis in zinc-deficient (*ZD*) mice. **A,** Schematic illustration of the experimental protocol. ZD and control mice were treated with both a ZD diet and an NADPH oxidase inhibitor, apocynin (300 mg/kg/d), in drinking water from 3 weeks of age. Next, the mice had undergone unilateral hindlimb surgery at 10 weeks of age. **B,** Quantitative analysis of the ischemic/nonischemic laser Doppler blood flow (LDBF) ratio in the ZD mice treated with apocynin at different points after surgery. **C,** Quantitative analysis of capillary density of the ischemic muscle in the ZD mice treated with apocynin at 14 days after surgery. Results presented as the mean ± standard deviation (n = 8 in each group). ∗*P* < .05. *NS,* Nonsignificant.

To examine whether excessive ROS production is involved in reducing the angiogenic capacity caused by zinc deficiency, the mice were treated with both a zinc-deficient diet and the NADPH oxidase inhibitor apocynin in their drinking water from 3 weeks of age (Fig 3, *A*). Next, the mice underwent unilateral hindlimb surgery at 10 weeks of age. Treatment with apocynin had partially restored the reduced angiogenic capacity caused by the zinc deficiency on day 14 after hindlimb surgery (Fig 3, *B*). Also, apocynin treatment partially restored the reduced capillary density caused by zinc deficiency (Fig 3, *C*).

### Association between zinc levels and SPP in patients with CLTI

Finally, we examined the relationship between the serum zinc levels and SPP as an index of tissue blood perfusion in patients with CLTI. The clinical characteristics of the patients are listed in Tables I and II. A total of 51 patients with CLTI who had undergone de novo revascularization were enrolled in the present study. The patients were aged 71.4 ± 8.1 years, and 66.7% were men. Their mean body mass index was 21.0 ± 4.4 kg/m^2^. The average serum zinc and copper levels were 62.3 ± 16.9 μ/dL and 129.3 ± 28.3 μ/dL, respectively. The mean levels of alkaline phosphatase, albumin, hemoglobin, and C-reactive protein were 292.8 ± 97.4 IU/L, 3.4 ± 0.6 g/dL, 11.0 ± 2.0 g/dL, and 2.8 ± 3.5 mg/dL, respectively. The proportions of smokers and those who required hemodialysis were 78.4% and 37.2%, respectively. The comorbidities included type 2 diabetes mellitus in 33 patients (57%), hypertension in 39 patients (78%), dyslipidemia in 27 patients (35%), CAD in 29 patients (41%), and cerebrovascular disease in 10 patients (24%). Using the Rutherford classification, 38 patients (40.9%) had had category 4 PAD, 51 (54.8%) had had category 5 PAD, and 51 (54.8%) had had category 6 PAD. The WIfI (wound, ischemia, foot infection) stage was as follows: stage 1, n = 4; stage 2, n = 6; stage 3, n = 10; and stage 4, n = 28. The mean SPP was 18.7 ± 8.9 mm Hg, and the average ankle brachial index was 0.45 ± 0.30. No patient with CLTI had required a primary major amputation.

**Table I:** Patient characteristics (n = 51)

Characteristic	Value
Age, years	71.4 ± 8.1
Gender	
Male	34
Female	17
Body mass index, kg/m^2^	21.0 ± 4.4
Serum zinc concentration, μg/dL	62.3 ± 16.9
Serum copper concentration, μg/dL	129.3 ± 28.3
Alkaline phosphatase, IU/L	292.8 ± 97.4
Albumin, g/dL	3.4 ± 0.6
Hemoglobin, g/dL	11.0 ± 2.0
C-reactive protein, mg/dL	2.8 ± 3.5
Hypertension	39
Diabetes mellitus	33
Hemodialysis	19
Dyslipidemia	27
Smoker	40
Coronary artery disease	29
Cerebrovascular disease	10
Ankle brachial index	0.45 ± 0.30
Skin perfusion pressure	18.7 ± 8.9
Drug	
α-Blocker	4
β-Blocker	17
Calcium blocker	31
Angiotensin II receptor blocker	18
ACE inhibitor	3
Diuretic	6
Cilostazol	25
Clopidogrel	19
Aspirin	36
Warfarin	9
Direct oral anticoagulant	1
Statin	21
Insulin	6
Prednisolone	4

*ACE,* Angiotensin-converting enzyme.

Data presented as numbers for categorical data or the mean ± standard deviation for continuous data.

**Table II:** Patient classification and treatment (n = 51)

Characteristic	No. (%)
Rutherford classification	
4	5 (10)
5	38 (74)
6	8 (16)
WIfI clinical stage	
1	2 (4)
2	8 (16)
3	17 (33)
4	24 (47)
GLASS stage	
1	2 (4)
2	2 (4)
3	47 (92)
Treatment	
Bypass surgery	34 (67)
Endovascular surgery	2 (4)
Bypass and endovascular surgery	15 (29)

*GLASS,* Global limb anatomic staging system; *WIfI,* wound, ischemia, foot infection.

**Fig 4:**
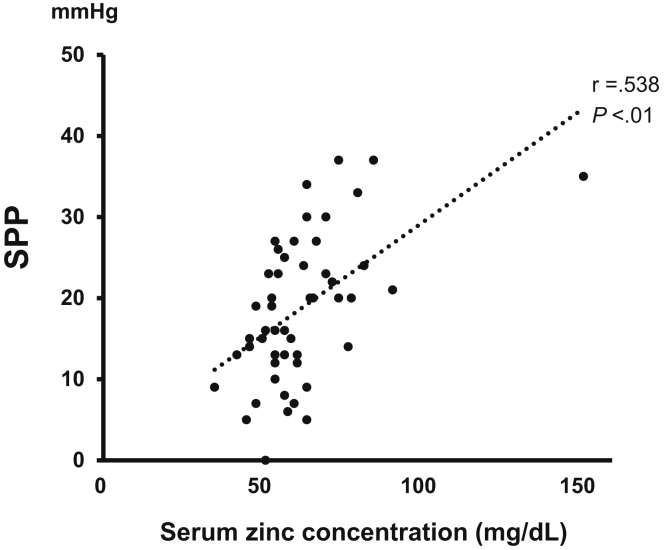
Association of serum zinc level with skin perfusion pressure (SPP) as an index of tissue blood perfusion in patients with chronic limb-threatening ischemia (CLTI). A total of 51 patients with CLTI who had been referred for de novo revascularization were enrolled in the present study. The results are presented as the mean ± standard error.

We performed univariate linear regression analysis to examine the relationship between the SPP and the clinical parameters of the patients with CLTI. The serum zinc levels were positively associated with the SPP (*r* = 0.538; *P* < .001; Table III and Fig 4). The albumin levels and the prevalence of diabetes, but not the copper levels, were also associated with the SPP (Table III). The oral administration of statins, antiplatelet agents, and antihypertensive agents was not associated with the SPP. Multiple linear regression analysis revealed that the serum zinc levels correlated positively with the SPP (*P* = .001; Table III). Thus, the serum zinc level was an independent predictor of the SPP as an index of tissue blood perfusion in patients with CLTI.

**Table III:** Correlation with skin perfusion pressure

Variable	Univariate		Multivariate	
	ρ	*P* value	β	*P* value
Serum zinc level	0.538	**.000**	0.445	**.001**
Albumin	0.302	**.031**	0.147	.265
Diabetes mellitus	0.288	**.041**	0.240	.058
Hemoglobin	0.255	.071	NA	NA
Age	−0.195	.171	NA	NA
Body mass index	0.194	.173	NA	NA
Antiplatelet count	0.193	.174	NA	NA
C-reactive protein	−0.191	.179	NA	NA
Hemodialysis	0.178	.212	NA	NA
Statin	0.163	.254	NA	NA
Cerebrovascular disease	0.158	.268	NA	NA
Antihypertensive agents	0.147	.302	NA	NA
Hypertension	0.082	.568	NA	NA
Female gender	0.061	.671	NA	NA
Smoker	−0.047	.743	NA	NA
Alkaline phosphatase	0.042	.772	NA	NA
Coronary artery disease	0.024	.865	NA	NA
Dyslipidemia	−0.017	.904	NA	NA
Serum copper level	0.008	.956	NA	NA

*NA,* Not applicable.

Boldface *P* values represent statistical significance.

## Discussion

In the present study, the major findings were as follows. First, zinc deficiency affected the reduction in revascularization rates in response to tissue ischemia. Second, zinc deficiency led to an increase in ROS production. Third, an NADPH oxidase inhibitor ameliorated the zinc deficient-induced impairment of revascularization. Finally, the serum zinc levels were positively associated with the SPP in the patients with CLTI. Thus, our data suggest that zinc can function as a regulator of the vascular response to ischemia.

Therapeutic strategies to facilitate angiogenesis and collateral vessel formation in patients with vascular insufficiency such as CLTI are important for ischemic tissue salvage.^21^ Zinc supplementation could be potentially useful in the treatment of such diseases. In addition, mesenchymal stromal cells (MSCs) are well known for their ability to differentiate into a variety of cell lineages, secrete several cytokines, and, by implantation, exercise an angiogenic effect.^22^, ^23^, ^24^ MSC implantation has been performed for CLTI at some institutions, with favorable results reported.^22^^,^^24^ A recent study has demonstrated that zinc supplementation caused a marked attenuation in the rate of cell apoptosis, enhanced cell viability, and increased the expression of angiogenic cytokines, resulting in enhancement of the angiogenic effects associated with MSC implantation.^25^ These findings suggest that MSC implantation combined with zinc supplementation is likely to be highly effective for therapeutic angiogenesis in patients with CLTI.

Zinc plays an important role in the activation of antioxidant enzymes, elimination of oxygen-derived free radicals, and generation of free radical scavengers in various organs, including the blood vessels.^26^ The prophylactic administration of zinc resulted in decreasing rates of oxidative stress and increasing glutathione peroxidase and superoxide dismutase enzyme expression in a cerebral hypoxia-ischemia rat model.^27^ In addition, zinc deficiency exacerbated the severity of pressure ulcers (PUs) in a cutaneous ischemia–reperfusion injury mouse model and increased the degree of cutaneous ischemia–reperfusion injury–induced vascular damage, oxidative stress, and apoptosis.^12^ Oral zinc supplementation also improved the severity of zinc deficiency-associated PUs.^12^ Clinically, zinc supplementation increased the enzymatic activity of superoxide dismutase enzyme in the blood in patients with type 2 diabetes mellitus, suggesting that it might be useful for patients with PUs.^28^ Consistent with these observations, we have demonstrated that zinc deficiency impaired the rate of ischemia-induced angiogenesis and increased the ROS levels in the ischemic muscles and the blood stream. Furthermore, an NADPH oxidase inhibitor ameliorated the zinc deficiency-induced impairment of revascularization. Thus, increased ROS production rates might adversely affect the angiogenic actions of zinc deficiency under ischemic conditions.

A previous study reported an association between low serum zinc levels and an increased prevalence of CAD. Among patients who have undergone coronary catheterization, the serum levels of zinc were significantly lower in the patients with CAD than in those with normal angiographic findings.^29^ In addition, the serum zinc/urine zinc ratio had an inverse correlation with CAD.^30^ In the present study, the serum zinc levels were positively associated with the SPP in patients with CLTI. In addition, we have previously shown that zinc deficiency was associated with lower rates of limb salvage in CLTI patients who had undergone bypass surgery.^10^ These data suggest that zinc levels might reflect the severity of CLTI. Taken together, the circulating zinc level might be not only a functionally important factor for the modulation of angiogenesis under pathologic conditions but also a useful marker for the assessment of atherosclerosis-based vascular disease such as limb ischemia.

In our preliminary data, the serum zinc levels did not significantly correlate with the albumin levels or the controlling nutritional status score, which is one of the nutritional indexes, in patients with CLTI. In the present study, a multivariate analysis that included the albumin levels revealed that the SPP correlated strongly with the serum zinc levels alone. Although malnutrition is a factor that reduces the zinc levels,^5^ our results have suggested that the various factors associated with the pathology of CLTI can affect the blood zinc levels more than will the patient's nutritional status.

In the various guidelines that have been reported for zinc deficiency, zinc deficiency has been defined as a serum zinc level of <60 μg/dL and marginal zinc deficiency as a serum zinc level of 60 to 80 μg/dL.^31^^,^^32^ In the present study, the mean zinc level in the patients with CLTI tended to be as low as 62.3 ± 16.9 μg/dL. It might be preferable for patients with CLTI to actively consume foods with a high zinc content, such as oysters and pork liver. Owing to the ease of measurement, the zinc levels should be regularly measured in patients with CLTI. If the serum zinc levels are extremely low, it might be beneficial to consider treatment with zinc adjuvants.

The present study had several limitations. First, in the present study, we measured the serum zinc levels at only one point and did not evaluate the subsequent changes over time. Second, we did not examine whether zinc supplementation could enhance the angiogenic capacity or improve the prognosis of the patients with CLTI. We believe that future studies are required to evaluate the effects of long-term zinc administration. Third, from an ethical perspective, we could not evaluate angiogenesis by performing immunostaining with muscle tissue samples collected from the patients with CLTI. Thus, in the present study, we were unable to directly evaluate the association between the serum zinc levels and angiogenic capacity in humans. Finally, owing to the small sample size, we were unable to examine how the different therapeutic strategies used for the patients with CLTI affected the prognosis in the present study.

## Conclusions

Zinc deficiency impaired the rate of revascularization in response to tissue ischemia, which is accompanied by oxidative damage. Nutritional approaches aimed at the intake of zinc might be useful for the prevention and/or treatment of ischemic limb disease. In addition, the preventive effects and usefulness of zinc require further investigation in largescale studies in the future.

## Author contributions

Conception and design: T Murohara, KK, RS

Analysis and interpretation: TT, A Kodama, SY, T Masutomi, RS

Data collection: TT, A Koyama

Writing the article: TT, KK, RS

Critical revision of the article: A Kodama, SY, T Masutomi, A Koyama, T Murohara

Final approval of the article: TT, A Kodama, SY, T Masutomi, A Koyama, T Murohara, KK, RS

Statistical analysis: TT, RS

Obtained funding: Not applicable

Overall responsibility: KK
