# Supplementary material for: Zinc deficiency impairs ischemia-induced angiogenesis
Source: JVS Vasc Sci. 2021 Dec 8;3:30–40. doi: 10.1016/j.jvssci.2021.09.023 (PMC8792263; doi:10.1016/j.jvssci.2021.09.023)
Supplement: Supplementary Material [file mmc1.pptx]

## Slide 1
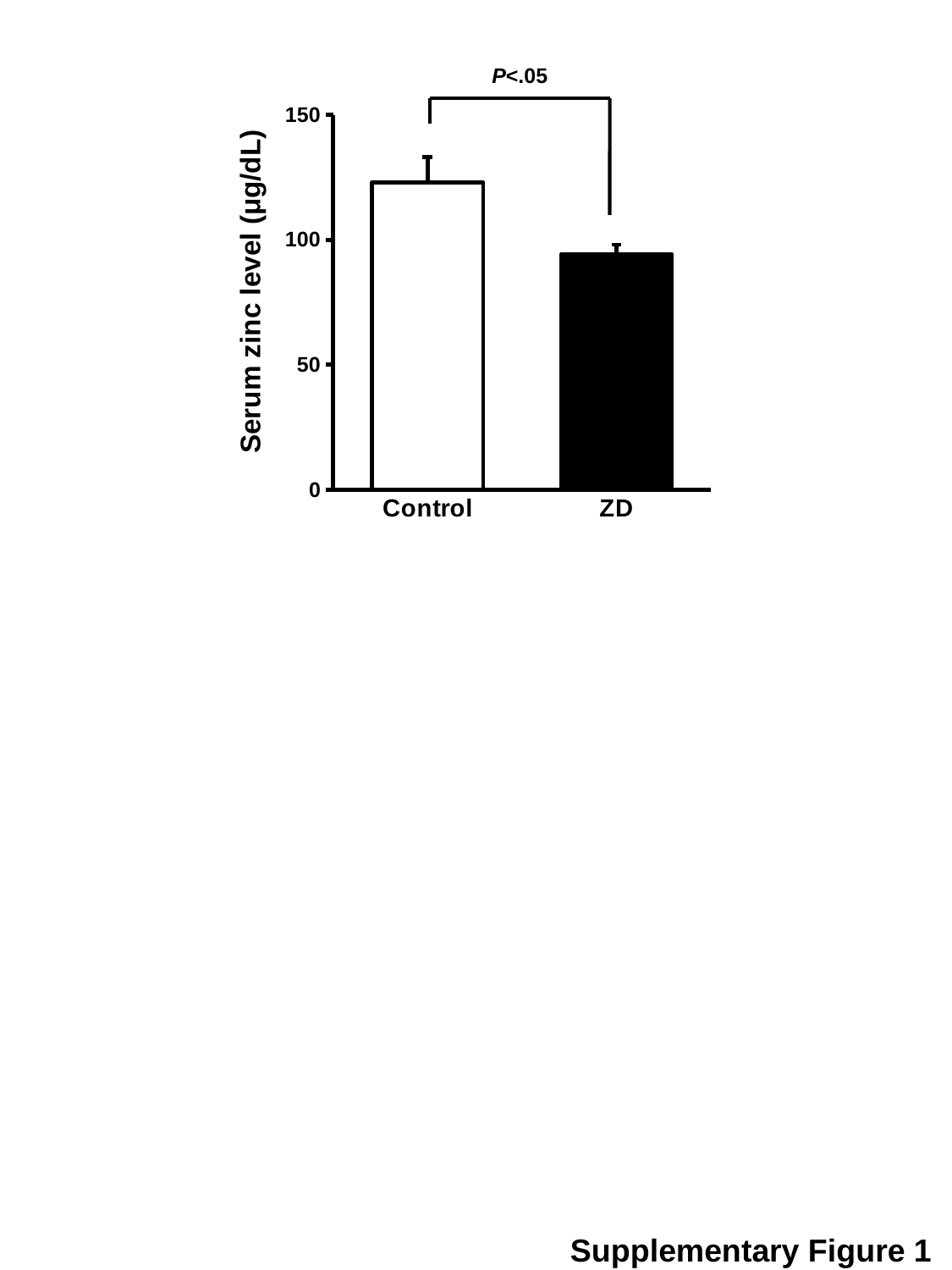

P<.05
### Chart
| Category | Serum Zinc concentration |
|---|---|
| Control | 123.00000000000001 |
| ZD | 94.225 |Serum zinc level (μg/dL)
Supplementary Figure 1

## Slide 2
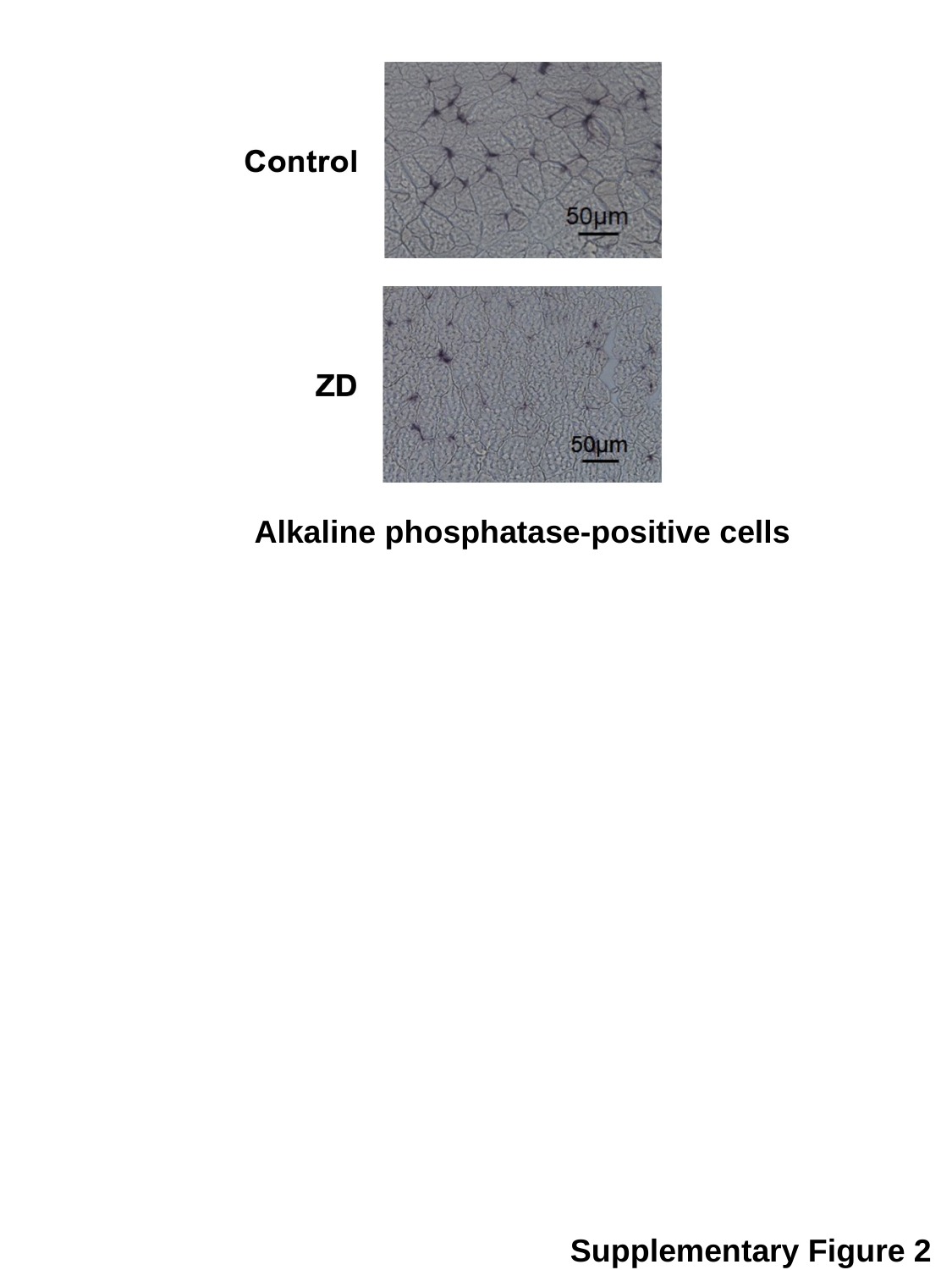

Alkaline phosphatase-positive cells
Supplementary Figure 2
